# Systems biology of angiogenesis signaling: Computational models and omics

**DOI:** 10.1002/wsbm.1550

**Published:** 2021-12-30

**Authors:** Yu Zhang, Hanwen Wang, Rebeca Hannah M. Oliveira, Chen Zhao, Aleksander S. Popel

**Affiliations:** ^1^ Department of Biomedical Engineering Johns Hopkins University School of Medicine Baltimore Maryland USA; ^2^ School of Pharmacy Nanjing Medical University Nanjing Jiangsu China

**Keywords:** angiogenesis, endothelial cell, mathematical model, multiscale systems biology, omics, signal transduction

## Abstract

Angiogenesis is a highly regulated multiscale process that involves a plethora of cells, their cellular signal transduction, activation, proliferation, differentiation, as well as their intercellular communication. The coordinated execution and integration of such complex signaling programs is critical for physiological angiogenesis to take place in normal growth, development, exercise, and wound healing, while its dysregulation is critically linked to many major human diseases such as cancer, cardiovascular diseases, and ocular disorders; it is also crucial in regenerative medicine. Although huge efforts have been devoted to drug development for these diseases by investigation of angiogenesis‐targeted therapies, only a few therapeutics and targets have proved effective in humans due to the innate multiscale complexity and nonlinearity in the process of angiogenic signaling. As a promising approach that can help better address this challenge, systems biology modeling allows the integration of knowledge across studies and scales and provides a powerful means to mechanistically elucidate and connect the individual molecular and cellular signaling components that function in concert to regulate angiogenesis. In this review, we summarize and discuss how systems biology modeling studies, at the pathway‐, cell‐, tissue‐, and whole body‐levels, have advanced our understanding of signaling in angiogenesis and thereby delivered new translational insights for human diseases.

This article is categorized under:

Cardiovascular Diseases > Computational Models
Cancer > Computational Models

## Graphical abstract

Multiscale and multicellular regulation of angiogenesis signaling (created with BioRender.com).

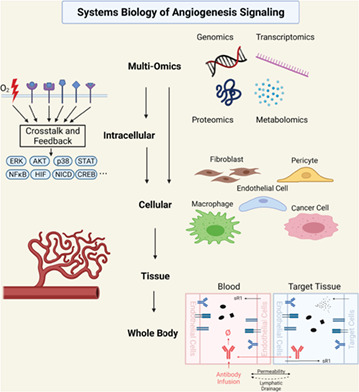

## 1 INTRODUCTION

Angiogenesis, the formation of new blood vessels from preexisting vessels, is a quintessential biological process widely involved in development, growth, wound healing and reproduction (Carmeliet & Jain, 2011; Eelen et al., 2020; Flegg et al., 2020; Logsdon et al., 2014). Angiogenesis by nature is a multiscale process and is tightly regulated by many molecular and cellular mechanisms and mediators (De Palma et al., 2017). For example, tissues experiencing lack of oxygenation can initiate hypoxia response via the intracellular hypoxia‐inducible factor (HIF) pathway and potently induce angiogenesis (de Heer et al., 2020; Semenza, 2012). Endothelial cells, among the many cellular regulators of angiogenesis, drive angiogenesis through processes including cell proliferation, migration, tip‐stalk cell selection, and cellular signaling which controls vascular permeability and stability (W. Chen et al., 2019; Gaengel et al., 2009; Lamalice et al., 2007). In complex tissue microenvironments in human diseases (e.g., cancer, ischemic diseases), a spectrum of biological signals is systematically integrated to determine the degree of angiogenic activities, which ultimately contributes to disease progression and resolution (Beck & Plate, 2009; De Palma et al., 2017; Johnson et al., 2019).

Dysregulation of angiogenesis is a phenomenon widely observed and implicated in the pathophysiology of various major human diseases. Carmeliet identified over 70 angiogenesis‐dependent diseases (Carmeliet, 2005) and the list has been growing. In tumors, excessive and abnormal angiogenesis (e.g., disorganized, leaky vessels) is critically involved in tumor growth and metastasis (Carmeliet & Jain, 2011). In the eye, pathological angiogenesis is an important contributing factor to vision impairment in many ocular diseases such as diabetic retinopathy and age‐related macular degeneration, affecting millions worldwide (Campochiaro, 2013). In ischemic diseases such as coronary artery disease (CAD), peripheral arterial disease (PAD), myocardial infarction (MI), and cerebral ischemia, insufficient formation of stable and nonleaky blood vessels is a pivotal reason behind the persistent damage and lack of natural regeneration of the ischemic tissue (Dragneva et al., 2013; Iyer & Annex, 2017; Yoo & Kwon, 2013). In infectious diseases, which include COVID19, the role of angiogenesis and endothelial dysfunction has been well recognized; more generally, angiogenesis and its associated molecular factors are key players in the immune response (Osherov & Ben‐Ami, 2016; Smadja et al., 2021). In regenerative medicine and tissue engineering, creating potent vascular networks is a crucial element in the optimal application of implants (Rouwkema & Khademhosseini, 2016). Since angiogenesis is innately a multi‐pathway, multifactorial, and multiscale process, the systems biology modeling approach, which mechanistically integrates knowledge and data across scales and studies, is particularly useful to help decipher the complex molecular/cellular interactions and emergent pathway/network features that dynamically regulate angiogenesis in human health and disease (G. Liu et al., 2011; Logsdon et al., 2014). Here, we review examples of systems biology modeling efforts toward the quantitative integrative understanding and translational investigation of angiogenesis from the following four aspects: intracellular signal transduction pathways of quintessential angiogenesis modulators (e.g., HIFs, VEGFs, Angiopoietins, FGFs), multi‐pathway cell‐level players involved in angiogenesis (e.g., endothelial cells, macrophages), blood vessel sprouting and formation, and tissue‐/body‐level characterization of angiogenesis and its implications in human diseases. We also discuss efforts that utilize multi‐omics data‐driven approaches to decipher novel angiogenesis drivers and core angiogenesis‐related pathway networks. Our review emphasizes the mechanistic relevance and significance of these systems biology models in the multiscale pathophysiology of angiogenesis‐related human diseases as well as the translational and therapeutic insights delivered by such modeling efforts. One of the themes is on cancer as cancer progression involves a wide spectrum of complex nonlinear cell signaling and communication. Of note, while the emphasis is on the various signaling pathways, here we will not dive deeply into the important mechanotransduction phenomena that affect angiogenesis, as there are excellent reviews devoted to the subject (Flegg et al., 2020; Murfee et al., 2015).

## 2 MODELS OF MAJOR CELLULAR/INTRACELLULAR INDUCERS OF ANGIOGENESIS

In this section, we discuss mechanistic models built to better understand some key inducing pathways of angiogenesis, including intracellular regulation of HIF and eNOS as well as the signal transduction networks of several growth factor‐mediated receptor pathways. While studying the unknown nonlinear emergent properties of known biological networks are usually of primary interest to modeling researchers, it can certainly be envisioned that the systems biology modeling framework and strategy can also be integrated with omics‐based high‐throughput data to predict and identify new molecular regulations, mechanisms, and hypotheses.

### 2.1 HIF stabilization and HIF‐mediated cellular pathways in angiogenesis

Hypoxia is a key driver of angiogenesis, and HIFs are the master regulators in the cellular hypoxic response (Krock et al., 2011; Semenza, 2012). At the molecular level, functional HIFs are heterodimers composed of the α and β subunits, while the α subunits (e.g., HIF1α, HIF2α) would typically undergo oxygen‐dependent protein degradation in normoxia. The biological processes that regulate HIFα stabilization and degradation under different oxygen tensions include a number of biochemical reactions with various interacting molecular species forming a highly dynamic system, and translational therapeutic modulations of HIF activity by targeting these reactions/species have been extensively investigated as an angiogenesis‐based strategies in cancer and ischemic diseases (Majmundar et al., 2010). Below we discuss systems biology computational models specifically formulated to advance the understanding of the different mechanistic aspects of HIF stabilization and HIF‐mediated cellular pathways in angiogenesis (selected model information is also summarized in Table 1).

**TABLE 1:** Summary of selected computational systems biology models of major intracellular signaling pathways in angiogenesis, including HIF stabilization and HIF‐mediated cellular pathways, eNOS regulation, and growth factor‐mediated pathways

Pathway/axis modeled	Cell type modeled	Relevant disease areas	Summary of model objectives/findings	References
HIF stabilization and HIF‐mediated signaling pathways				
Hypoxia‐HIF1α	General	General	Showed that variations in intracellular molecular environment would result in different HIF1α expression patterns	(Qutub & Popel, 2006)
Hypoxia‐HIF1α	General/validated experimentally in HEK293 cells	General	Showed that the different mechanistic functions of PHD and FIH would result in a nonlinear relationship between HIF1α protein stability and its transcriptional activity	(Nguyen et al., 2013)
HIF‐microRNA‐VEGF	Endothelial cells	Cancer, PAD	Simulated cellular production of VEGF under hypoxia and microRNA regulation; proposed a mechanistic explanation for insufficient VEGF response in ischemic vascular diseases	(Zhao & Popel, 2015)
HIF‐TGFβ‐microRNA‐TSP1	Endothelial cells, fibroblast	Cancer, PAD	Simulated cellular TSP1 production under the control of hypoxia, TGFβ signaling and microRNAs; evaluated in silico strategies that can therapeutically modulate production of TSP1 under relevant pathological conditions	(Zhao et al., 2017)
HIF1α‐ROS	General	Cancer, ischemia	Explained how ROS affects HIF regulation through opposite mechanisms and the resulting differences in the HIF response in tumor and ischemia/reperfusion	(Qutub & Popel, 2008)
HIF1α‐p53	General	General	Characterized reciprocal mechanistic regulation between HIF1a and p53 under different hypoxic conditions	(P. Wang, Guan, et al., 2019)
Regulation of eNOS function				
VEGF/VEGFR2‐calcium‐eNOS/cGMP	Endothelial cells	Cancer	Simulated targeted interventions to inhibit eNOS activity under high tissue VEGF concentrations in tumor	(Q. Wu & Finley, 2020)
Insulin‐MAPK/endothelin1‐PI3K/eNOS	Endothelial cells	Diabetes	Characterized differential changes of eNOS and endothelin1 activities under different pathological conditions	(Muniyappa et al., 2020)
Shear stress‐eNOS	Endothelial cells	General	Characterized multilevel regulation of eNOS function under fluid shear stress and evaluated the impact of different interventional strategies	(Koo et al., 2013)
eNOS uncoupling	Endothelial cells	General	Investigated a wide spectrum of mechanisms relating to oxidative stress‐induced eNOS uncoupling and proposed model‐based strategies to restore eNOS function	(Joshi et al., 2017)
Growth factor‐mediated intracellular signaling pathways				
VEGF, sVEGFR1	Endothelial cells	PAD	Used a mechanistic model of sVEGFR1 to quantitatively explore its mechanism of acting as ligand trap and heteromerization with VEGFR	(F. T. Wu et al., 2009)
VEGF, sVEGFR1	Endothelial cells	General	Used a PDE‐based model of sVEGFR1 secretion and distribution to show its effect in coordinating blood vessel formation stages	(Chappell et al., 2016)
VEGF isoforms	Endothelial cells	PAD, general	Demonstrated the effect of the splice isoform of VEGF, VEGF165b's effect on VEGFR2 signaling through VEGFR1 interactions	(Clegg et al., 2017)
VEGF, TSP‐1	Endothelial cells	General	Using a rule‐based model to demonstrate the role of TSP‐1/CD47 interaction on VEGFR2 signaling and downstream activation of AKT/ERK and intracellular calcium	(Bazzazi et al., 2017; Bazzazi, Zhang, Jafarnejad, Isenberg, et al., 2018)
VEGFR‐Integrin	Endothelial cells	General	Used a rule‐based model of integrin‐VEGFR2 interaction to investigate potential mechanisms of integrin‐targeted therapies	(Bazzazi, Zhang, Jafarnejad, & Popel, 2018)
Ang‐Tie, sTie2	Endothelial cells	General	Simulated the effect of soluble Tie2 acting as a ligand trap of Ang1 and compared its effects with engineered ligand trap using an ODE‐based model	(Alawo et al., 2017)
Ang‐Tie	Endothelial cells	General	Used a mechanistically detailed models of Ang/Tie signaling pathway, its molecular mechanisms and junctional localization to identify potential mechanistic targets for Tie2‐targeted therapy	(Y. Zhang et al., 2019; Y. Zhang, Kontos, et al., 2021)
FGF2	General	General	Proposed a kinetic model of FGF and its complex formation with FGFR and HSPG	(Ibrahimi et al., 2004)
FGF‐FGFR	Endothelial cells	General	Used a PDE‐based model to demonstrate the effects of FGF triad formation and its intracellular responses	(Filion & Popel, 2004)
FGF‐2	Myocardium	Cardiovascular disease	Investigated the distribution and retention of FGF‐2 following exogenous FGF administration using a compartmental model	(Filion & Popel, 2005)
FGF‐2	General	General	Used a finite element model of FGF diffusion and reaction to simulate FGF binding kinetics under fluid flow	(Patel et al., 2013)
HGF	Hepatocytes	Cancer	Used a combination of quantitative and qualitative modeling to identify and validate a signaling network of HGF‐stimulated Akt and ERK activation	(D'Alessandro et al., 2015)
HGF	Cancer cells, cancer‐associated fibroblasts	Cancer	Developed a PDE‐based model of the HGF/c‐Met signaling between cancer‐associated macrophages and tumor cells in the tumor microenvironment	(Konstorum & Lowengrub, 2018)
HGF	Hepatocytes	Cancer	Tested the synergism of combination therapies targeting the HGF signaling axis with mechanistic model of the HGF/cMet signaling pathway	(Jafarnejad et al., 2019)

#### 2.1.1 Intracellular regulation of HIF1α by prolyl and asparaginyl hydroxylases

Physiological interaction between HIFα subunits and intracellular hydroxylases (e.g., PHDs, FIH) is a primary regulatory step for the subsequent recognition and targeted proteasomal degradation of HIFα by VHL‐containing ubiquitin ligase complexes. A series of computational models have been proposed and analyzed to study HIF stabilization (Nguyen et al., 2013). Among those, the work by Qutub et al. is the first mechanistic model that included kinetic details of PHD2 binding with its cofactors such as iron, ascorbate, 2‐oxoglutarate, and oxygen (Qutub & Popel, 2006). The model concluded that the hypoxia‐induced HIF1α response dynamics could vary significantly depending on different molecular compositions of those cofactors within the cell, which suggested that interventions targeting HIF1α hydroxylation to modulate HIF1α activity may also have different effectiveness across human cell lines. Later, the model developed by Nguyen et al. further incorporated details of FIH‐mediated HIF1α asparagine hydroxylation and negative feedback through HIF1‐mediated PHD production, together to describe the transcriptional activity of HIF1α under various physiological and pharmacological perturbations (Nguyen et al., 2013). Using their model, the authors predicted an unexpected biological feature regarding the diverging regulation of HIF1α stabilization and HIF1α transcriptional activity when both PHD and FIH are inhibited; further, this seemingly counterintuitive prediction was validated by their experimental results and was explained mechanistically by an emergent model‐based regulatory axis.

#### 2.1.2 Complex interactions between HIF1α and p53

The tumor suppressor protein p53 is a well‐known transcription factor that has wide impacts on cell cycle, apoptosis, senescence, DNA repair and metabolism (Joerger & Fersht, 2016). It is also stabilized under severe hypoxia and can participate in HIF regulation and be regulated by HIFs via different routes (Obacz et al., 2013). On this topic, two related computational models were developed to mechanistically describe the multimodal regulatory cross talk and feedback between HIF1α and p53 pathways under hypoxia (P. Wang, Guan, et al., 2019; Zhou et al., 2015). In the latter model, by performing model stability analyses, the authors proposed a potential bifurcation between predominant intracellular activation of p53 versus HIF1α based on different severities of hypoxia which might serve as an essential axis that regulates the dynamic and coordinated determination of cell fate and pro‐angiogenic activation during hypoxia.

#### 2.1.3 HIF1α‐ROS interactions

Reactive oxygen species (ROS), a class of oxygen‐based cellular metabolic products, can also participate in the regulation of HIFs. Experimental studies have shown that PHDs, FIH, and HIFs can be regulated by ROS through a number of direct and indirect mechanisms (reviewed in Acker et al., 2006; Wong et al., 2013). Using the systems biology approach, Qutub and Popel developed a computational model based on mass‐action kinetics and incorporated four possible mechanisms of HIF1α‐ROS interaction to explain some seemingly contradictory findings in literature on how ROS regulates HIF1α (Qutub & Popel, 2008). The authors analyzed the model simulations under various concentration combinations of five different molecular species that have functional ties to ROS‐HIF1α (O2, iron, ascorbate, succinate, 2‐oxoglutarate) and provided a mechanistic basis to better understand the differential HIF1α time‐course response influenced by ROS in tumors versus in non‐tumor ischemia.

#### 2.1.4 HIF‐mediated cellular production of pro‐ and antiangiogenic factors

Hypoxia, through the activation of HIFs and other transcription factors, has been shown to regulate the cellular synthesis and secretion of a number of pro‐ and anti‐angiogenic molecules (e.g., VEGF, Angiopoietins, MMPs, TSP1) to control angiogenesis (Krock et al., 2011). Our group has developed two computational models that investigated the dynamic cellular synthesis of VEGF and TSP1, respectively, under the coordinated control of hypoxia, cytokine signaling, and posttranscriptional regulation (e.g., by microRNAs; Zhao et al., 2017; Zhao & Popel, 2015). Driven by thorough quantitative calibration against time‐course experimental data as well as focused analyses using clinical and pathophysiological data, the models were then used to propose novel mechanistic explanations for the disrupted angiogenic balance in specific human diseases (e.g., suppressed TSP1 expression in tumors due to oncogenic activation of Myc, insufficient induction of VEGF in PAD calf muscle due to dysregulation of microRNA let‐7). Moreover, the models have been used to provide quantitative model‐based evaluation of various pathway‐targeted therapeutic interventions under disease‐relevant pathological conditions in vitro.

### 2.2 Regulation of eNOS function and its impact in the modulation of angiogenesis

Endothelial nitric oxide synthase (eNOS or NOS3) is the predominant producer of nitric oxide (NO), an important vasodilator and regulator of angiogenesis, in endothelial cells. Activation of eNOS is regulated by multiple growth factor‐driven pathways (e.g., VEGF, HGF, Insulin), calcium‐dependent pathways, as well as fluid shear stress (Fukumura et al., 2001; Koo et al., 2013; Makondo et al., 2003; Muniyappa et al., 2020). Below we discuss systems biology models that were developed to characterize the complex signaling network of stimulation‐induced eNOS functional activation and explore its potential therapeutic implications.

#### 2.2.1 eNOS activation regulated by VEGF/VEGFR2 and calcium

Using rule‐based modeling, Bazzazi et al. formulated the first mechanistic model that describes eNOS phosphorylation by the PI3K/AKT and calcium/calmodulin axes downstream of VEGF/VEGFR2 pathway (Bazzazi, Zhang, Jafarnejad, Isenberg, et al., 2018). Using the model, the authors tested the influence of the anti‐angiogenic matricellular protein thrombspondin‐1 (TSP1), which has been implicated in the pathophysiology of PAD and can associate with cellular CD47 to disrupt VEGF/VEGFR2 signaling, on VEGF‐induced eNOS phosphorylation. They demonstrated that eNOS activation, compared to AKT, is particularly sensitive to the inhibitory effect introduced by TSP1 and may require targeting of both CD47 and TSP1 to rescue its normal activation in angiogenesis (Bazzazi, Zhang, Jafarnejad, Isenberg, et al., 2018). Later, the work by Wu and Finley further advanced this rule‐based framework and incorporated the chaperone protein HSP90 as well as multiple activation states of eNOS and its downstream signal transduction involving arginine, NO, sGC (soluble guanylate cyclase) and cGMP (cyclic guanosine monophosphate; Q. Wu & Finley, 2020). They then analyzed the model under different VEGF concentrations that resembled actual VEGF levels present in the tumor microenvironment and evaluated the therapeutic potential of several new intracellular targets/processes in terms of shutting down VEGF‐induced pro‐angiogenic activation of eNOS and cGMP.

#### 2.2.2 eNOS activation regulated by shear stress

An integrated systems biology model has been developed by Koo et al. to investigate the multilevel regulation of eNOS induced by fluid shear stress from four different aspects: calcium influx and eNOS activation, AKT activation and eNOS phosphorylation, eNOS production, and functional eNOS complex formation (Koo et al., 2013). This mechanistic platform‐style model was able to describe time‐course experimental data of shear stress‐induced eNOS/NO production on the scales of minutes to hours. The model also enabled direct quantitative comparison of functional eNOS generated from two different routes (calcium versus phosphorylation) during shear stress stimulation and their relative contribution to NO production in endothelial cells. Furthermore, the authors also implemented model‐based interventions that target two different transcription factors as well as two different modalities that inhibit AKT and characterized their respective influences on shear stress‐induced eNOS activation. This is an example of model‐based quantitative characterization of mechanical stress‐induced endothelial cell signaling; more thorough discussion of multiscale modeling efforts applied toward a better understanding of the mechanical aspects of angiogenesis can be found in Flegg et al. (2020) and Murfee et al. (2015).

#### 2.2.3 eNOS uncoupling

In endothelial cells, oxidative stress can cause eNOS dysfunction (eNOS uncoupling) by depleting tetrahydrobiopterin (BH4), an essential cofactor for eNOS. This will shift eNOS from a NO‐producing enzyme to a superoxide‐producing enzyme, which would further potentiate the preexisting cellular oxidative stress and endothelial dysfunction (Luczak et al., 2020). To quantitively and dynamically describe the set of complex biochemical reactions governing the process of eNOS uncoupling, Kavdia's group, through a series of systems biology studies, has developed a comprehensive computational model that mechanistically incorporated molecular‐level interactions between eNOS, BH4, oxidized biopterins, SOD (superoxide dismutase), L‐arginine, oxygen, NO, CO2, ROS, and reactive nitrogen species (Joshi et al., 2017; Kar et al., 2012; Kar & Kavdia, 2011). Using this model, the authors unveiled that as oxidative stress increases beyond a threshold level, eNOS can transition from the coupled state into an uncoupled state accompanied with oscillations of decreased NO production over time. Model analyses also suggested that BH4 supplementation combined with strategies to reduce oxidative stress would be an optimal therapeutic approach to improve eNOS coupling and endothelial dysfunction in diseases (Joshi et al., 2017).

### 2.3 Growth factor‐mediated signaling pathways that drive angiogenesis

Growth factors are secreted protein molecules that bind and activate receptors to regulate physiological processes including angiogenesis. Growth factor‐mediated intracellular signaling pathways control various aspects of angiogenesis, including vascular permeability, maturation, quiescence, and stability (Potente et al., 2011). Growth factors and their receptors are regulated by a variety of molecular mechanisms, including ligand secretion and expression, receptor multimerization, ligand competition, receptor trafficking and turnover, co‐receptor interactions, downstream regulation of receptor expression, and many other pathway specific regulatory mechanisms (Lemmon & Schlessinger, 2010). The growth factor/receptor interactions, their regulatory mechanisms, and downstream signaling form complex reaction networks that warrant the use of integrative computational systems biology models to gain quantitative understanding of the behavior of the signaling pathway. Below we discuss selected computational models of growth factor‐mediated signaling pathways (summarized in Table 1). It should be noted that some of the studies of FGF and HGF pathways described below were conducted in application to non‐endothelial cells, for example, fibroblasts and hepatocytes; however, the models are applicable to endothelial cells, with appropriate calibration.

#### 2.3.1 VEGF

The vascular endothelial growth factor (VEGF) signaling pathway is a major signaling pathway regulating vascular growth, maintenance, and remodeling. The VEGF signaling pathway also mediates calcium signaling patterns in endothelial cells associated with proliferation and migration (Noren et al., 2016). The VEGF ligand‐receptor system consists of ligands VEGF‐A, VEGF‐B, VEGF‐C, VEGF‐D, and placental growth factor (PlGF), receptors VEGR1, VEGFR2, and VEGFR3, and co‐receptors neuropilins NRP‐1 and NRP‐2 (Mac Gabhann & Popel, 2008). In turn, the alternative slicing of VEGF‐A gives rise to at least 16 different isoforms with distinct signaling properties, as reviewed in Peach et al. (2018). VEGF signaling pathway is also regulated by TSP‐1 and its interaction with CD47 (Kaur et al., 2014), expression of the soluble VEGF receptor 1 (sVEGFR1/sFLT1; F. T. Wu et al., 2010a), splicing isoforms and extracellular regulation of VEGF (Vempati et al., 2014), as well as integrin binding (Somanath et al., 2009). The complexity of the signaling pathway and its myriad of regulatory mechanisms have given rise to computational models that focus on different aspects of the pathway, reviewed in Finley et al. (2015) and Logsdon et al. (2014). F. T. Wu et al. (2009) and Chappell et al. (2016) used ODE‐based and PDE‐based models, respectively, to quantitatively explore the effect of sVEGFR1 in pathological states and in different stages of blood vessel formation. Wu et al. used a compartmental model of VEGF, sVEGFR1, and their interaction with VEGFR2 to investigate the mechanisms of sVEGFR1's inhibition of VEGF signaling, including it acting as a ligand trap and its heterodimerization with VEGFR (F. T. Wu et al., 2009). Chappell et al.'s model incorporated both inhibitory mechanisms of sVEGFR1 in an integrated PDE‐based model of VEGF and VEGFR interaction that demonstrates the stage‐specific effects of VEGF during different stages of vascular morphogenesis (Chappell et al., 2016). Clegg et al.'s model focused on the splice isoform VEGF165b and its effect on VEGFR2 signaling through competition of binding to VEGFR1 (Clegg et al., 2017). Mac Gabhann et al. used an ODE‐based model, combined with experimental validation, to demonstrate that heterodimers of VEGFR1 and VEGFR2 form at the expense of homodimers at the cell surface, resulting in distinct downstream signaling transduction activities (Mac Gabhann & Popel, 2007). In a more integrative model, Bazzazi et al. used rule‐based modeling to construct the complex reaction networks formed by VEGF, its receptors and co‐receptor, the interaction with TSP1/CD47, and downstream signaling through AKT, ERK, and intracellular calcium to demonstrate the potential effects of targeting the TSP1/CD47 axis, and the potential effectiveness of combination of pro‐angiogenic therapies to rescue VEGF signaling to Akt and eNOS involving this strategy (Bazzazi et al., 2017; Bazzazi, Zhang, Jafarnejad, Isenberg, et al., 2018). In another model, Bazzazi et al. also investigated the potential molecular mechanisms of the interaction of αVβ3 integrin and VEGFR2 and predicted the effects of integrin‐targeting therapeutics in inhibiting angiogenesis (Bazzazi, Zhang, Jafarnejad, & Popel, 2018).

#### 2.3.2 Ang/Tie

The Angiopoietin (Ang)/Tie signaling pathway is a major endothelial signaling pathway regulating vascular permeability, stability, and quiescence (Eklund & Saharinen, 2013). The Ang/Tie ligand receptor system consists of angiopoietins Ang1‐4, receptor Tie2, and co‐receptor Tie1 (Leppanen et al., 2017), and is regulated by receptor multimerization (Kim et al., 2005), phosphatase VE‐PTP (Winderlich et al., 2009), receptor trafficking and junctional localization (Saharinen et al., 2008), and receptor extracellular domain cleavage (Singh et al., 2012). Computational systems biology modeling allows the complex integration of reaction network formed by the interactions of Ang1 and Ang2 with Tie2, and the molecular regulatory mechanisms. The extracellular domain of Tie2 is known to be cleaved off from the surface of the endothelial cells in inflammatory conditions, forming soluble form of the receptor that can bind and inhibit Ang ligands (Findley et al., 2007). Alawo et al. developed an ODE‐based computational model to simulate the effect of soluble Tie2 and an engineered ligand trap on the inhibition of Tie2's activation (Alawo et al., 2017). Our group has also developed ODE‐based models, using rule‐based modeling, of the Ang/Tie signaling pathway that included detailed molecular mechanisms such as receptor multimerization and clustering, ligand competition and co‐receptor binding, receptor trafficking, turnover, extracellular domain cleavage and degradation, junctional localization and downstream signal transduction, and regulation by the VE‐PTP (Y. Zhang et al., 2019; Y. Zhang, Kontos, et al., 2021). The models identified that the expression of VE‐PTP, the presence of Tie1, and its junctional localization are key molecular events modulating the context‐dependent agonistic function of Ang2 and provided a mechanistically detailed mechanism to resolve the controversial roles of Tie1 on Tie2's activation. The models also identified inhibiting the extracellular domain cleavage of Tie2, inhibiting VE‐PTP, and promoting Tie1's junctional localization as potential therapeutic strategies to promote vascular stability.

#### 2.3.3 FGF

The fibroblast growth factor (FGF) signaling pathway, consisting of FGF‐2 and receptors FGFR1‐4, is another important signaling pathway regulating angiogenesis, wound healing, and tissue repair (Bikfalvi et al., 1997). The FGF signaling pathway is regulated by the release pattern of FGF, heparan sulfate proteoglycans (HSPGs), and complex formation between FGF, FGFR, and HSPGs (Bikfalvi et al., 1997). Ibrahimi et al. proposed an ODE‐based, kinetic model of the FGF‐2/FGFR1/HSPG complex assembly based on surface plasmon resonance data (Ibrahimi et al., 2004). Filion et al. used a PDE‐based reaction–diffusion model of FGF‐2 and its interaction with cell surface receptors to demonstrate the effect of FGF‐2 dimerization and the assembly of triads (FGF‐2/FGFR1/HSPG) and double triads (2 FGF‐2/FGFR1/HSPG) on the intracellular signaling response of the FGF signaling pathway (Filion & Popel, 2004). Another model by Filion et al. used a compartmental model to investigate the effect of distribution and retention of exogenous FGF‐2 on its bioavailability (Filion & Popel, 2005). Patel et al. used a finite‐element based model to simulate the different dynamics of FGF‐2/FGFR/HSPG triad and FGF‐2/HSPG or FGF‐2/FGFR complex formation following continuous and bolus delivery of FGF‐2 under varying flow conditions, and examined the effects of binding stoichiometry, binding site density, fluid flow, delivery dose, and delivery mode to inform experimental studies on FGF‐2 delivery (Patel et al., 2013). In addition to the role of FGF signaling in angiogenesis, other computational models have also been developed to study the biphasic dose–response of FGF‐2 due to the its complex formation dynamics with FGFR and HSPG (Kanodia et al., 2014), as well as its potency in activating ERK signaling in fibroblasts compared to platelet‐derived growth factor (PDGF; Cirit & Haugh, 2012).

#### 2.3.4 HGF

Additional signaling pathways, including the hepatocyte growth factor (HGF) signaling pathway, has been demonstrated to play an important role in angiogenesis. Its downstream signaling has been demonstrated to be able to stimulate endothelial proliferation, migration, and vascular morphogenesis (Shojaei et al., 2010). HGF signaling pathway consists of HGF and its receptor, c‐Met, and often acts synergistically with the VEGF signaling pathway (You & McDonald, 2008). D'Alessandro et al. used a hybrid approach utilizing both quantitative experimental data and qualitative molecular interaction graph to systematically identify and validate the best model structure of HGF‐stimulated signaling of Akt and ERK activation, predicting that the efficient inhibition of the downstream signaling of HGF can be achieved by targeting the combinations of PDK1, PI3k, Met, and ERK (D'Alessandro et al., 2015). Konstorum and Lowengrub used a PDE‐based, multispecies model of the tumor microenvironment to simulate the interaction of HGF‐secreting cancer‐associated fibroblasts with c‐Met receptors on tumor cells, predicting that disrupting the HGF/c‐Met signaling with anti‐HGF or anti‐c‐Met therapy reduces tumor invasiveness and growth (Konstorum & Lowengrub, 2018). Jafarnejad et al. used an ODE‐based, mechanistic computational model of HGF, c‐Met, its interaction with integrin, and downstream signaling to simulate the effects of monotherapies and combination therapies targeting the HGF signaling axis (Jafarnejad et al., 2019). The model incorporated omics data to predict patient‐specific synergism and antagonism of different combination therapies, identified the potential synergistic efficacy of the simultaneously targeting integrin and HGF, Met, or Raf in hepatocellular carcinoma, and provided a framework to identify patients who could benefit from drug combinations with mRNA expression data (Jafarnejad et al., 2019). It should be noted that the models discussed above focus on hepatocytes and cancer cells but can be adapted to other cell types such as endothelial cells with proper calibration.

## 3 MULTI‐PATHWAY MODELS OF ANGIOGENESIS DRIVER CELLS

Cellular signaling pathways that regulate angiogenesis often interact with each other through signal competition, redundancy, shared downstream signaling network, and many cross talk and cross‐regulation mechanisms. Models of individual signaling pathways cannot capture important cellular behaviors as a result of cross‐pathway interactions. In this section, we summarize computational models at the cellular level that integrate multiple cellular signaling pathways in major endothelial driver cells, including endothelial cells, macrophages, cancer cells, and fibroblasts.

The orchestration and regulation of angiogenesis also require communication of endothelial cells with other angiogenesis driver cells. Understanding the intercellular interactions during angiogenesis beyond the cellular level requires computational models that integrate multiple cell types. Below we also discuss examples of computational models at the multicellular level that focus on cell–cell communication across different cell types in angiogenesis.

### 3.1 Endothelial cells

Endothelial cells are major driver cells of angiogenesis. They participate in and regulate key steps of angiogenesis, including tip/stalk cell selection (Jakobsson et al., 2010), sprouting angiogenesis, elongation, and maturation, and regulation of vascular permeability and quiescence (Eilken & Adams, 2010; Lamalice et al., 2007). In the previous section, we have discussed computational models of intracellular signaling pathways. In endothelial cells, multiple intracellular pathways can interact and regulate each other, and have cross talk mechanisms that form integrative reaction networks that encompass several signaling pathways. Here we summarize computational models that have been developed to simultaneously simulate multiple intracellular signaling pathways in endothelial cells.

In the hybrid model of endothelial cell rearrangement during sprouting angiogenesis, Song and Finley used ODE‐based models of the VEGF and FGF signaling pathways to study the effects of simultaneous stimulation by VEGF and FGF on their shared downstream signaling pathways through AKT and ERK (Song & Finley, 2018, 2020). The authors quantitatively demonstrated that compared to targeting either VEGF or FGF in isolation, simultaneous targeting both pathways showed potential synergism, resulted in higher efficacy, and a faster and more sustained response in inhibiting the activation of ERK, informing the development of combination anti‐angiogenic therapies targeting both pathways in cancer treatment (Song & Finley, 2018). Bauer et al. used a Boolean network of growth factor/receptor tyrosine kinase pathway, cadherin signaling, integrin signaling, and their downstream signaling pathways to perform in silico investigations of signaling cross talk and identify therapeutic targets for modulating endothelial cell migration, proliferation, apoptosis, and quiescence (Bauer et al., 2010). Weinstein et al. constructed a Boolean molecular regulatory network of angiogenesis control that incorporates multiple signaling pathways (including VEGF, Ang/Tie, HGF, IGF), their downstream signaling, and the mechanosensory mechanisms to study the effect of the endothelial microenvironment during angiogenesis on a network level (Weinstein et al., 2017). The model is further extended in a subsequent study to include the molecular regulatory network in endothelial‐to‐mesenchymal transition (EndMT) that encompasses pericytes, tip and stalk endothelial cells, and mesenchymal cells to investigate the extracellular microenvironment and the molecular activation patterns involved in EndMT (Weinstein et al., 2020).

**FIGURE 1:**
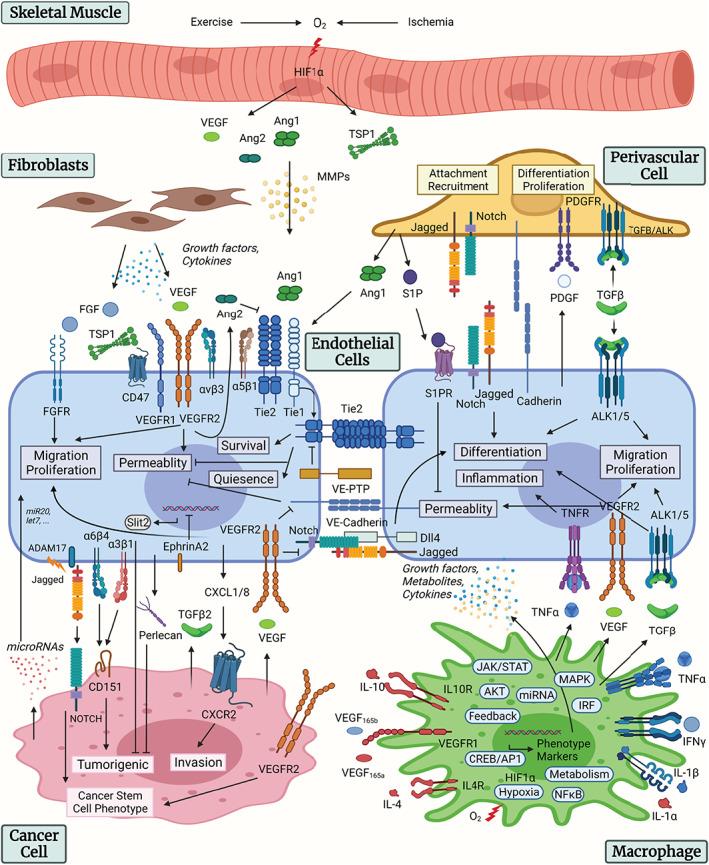
Overview of endothelial signaling and cell–cell communication. Summary of the intracellular signaling pathways of the endothelial cells and the cell–cell communication mechanisms with pericytes, macrophages, fibroblasts, cancer cells, and skeletal myocytes. Figure created with BioRender.com

Most endothelial phenotypes are simultaneously controlled by multiple signaling pathways. Endothelial permeability in microvasculature is simultaneously regulated by the VEGF and Ang/Tie signaling, VE‐cadherin signaling, and other signaling mechanisms (Lampugnani et al., 2018). One mechanism by which VEGF promotes vascular permeability is through promoting phosphorylation and subsequent internalization of VE‐Cadherin via Src kinase (Wallez et al., 2007). Angiopoietins, through activating Tie2, can inhibit VEGF‐induced permeability by sequestering Src kinase through its downstream signaling pathway (Gavard et al., 2008). VEGF and Ang/Tie signaling pathway also interact with and regulate each other through a variety of cross talk mechanisms (Fiedler et al., 2004; Findley et al., 2007) to form an integrative reaction network. Quantitative understanding of the integrative signaling network formed by multiple pathways therefore requires multi‐pathway models that simultaneously simulate these pathways and their cross talk on a systems level. The models can also guide experimental studies and the search for therapeutic strategies that target multiple pathways. The major endothelial signaling pathways regulating angiogenesis are summarized in Figure 1. The figure also illustrates endothelium‐centric cell–cell interactions in the tissue microenvironment with the following cell types included: macrophage, fibroblast, pericyte, cancer cell, and skeletal myocyte. Below we describe the relevant signaling models.

### 3.2 Macrophages

Macrophages are functionally versatile immune cells that are crucially involved in a number of biological processes, including angiogenesis (Corliss et al., 2016). They can be programmed by the wide range of signals in the tissue microenvironment to dynamically shape their phenotypes and functions, a process known as macrophage polarization. The full spectrum of macrophage polarization can be characterized as a multidimensional continuum of vastly diverse phenotypes with the canonical M1–M2 notions referring to its two extremes (Sica & Mantovani, 2012). Generally, M1‐like macrophages are pro‐inflammatory and tumoricidal, while M2‐like macrophages have been associated with high anti‐inflammatory, pro‐tumorigenic, and pro‐angiogenic potential as revealed by numerous in vitro and in vivo studies (reviewed in P. Chen & Bonaldo, 2013; Corliss et al., 2016). In order to integratively characterize the underlying dynamics that govern macrophage polarization in physiology and explore its therapeutic potential (e.g., promoting angiogenesis) in human diseases, computational systems biology modeling is an ideal approach given the large number of involved signal transduction pathways, feedback and cross talk mechanisms, phenotype markers, and effector molecules.

Implementing this idea, several multi‐pathway computational models of macrophages have been developed in the past 5 years using different approaches to investigate the general cell‐level polarization processes (including considerations of how macrophages contribute to angiogenesis). Three computational models by Palma et al. (2018), Ramirez et al. (2019), and Marku et al. (2020) used semi‐mechanistic Boolean network approaches to identify activation patterns of macrophages under simplified M1–M2 networks formulated by direct protein–protein interactions. Another two computational models by Rex et al. (2016) and X. Liu et al. (2021), respectively, used a combination of logic‐based modeling and deterministic ordinary differential equations (ODEs) to simulate M1–M2 macrophage marker expression signatures induced by an array of pathophysiological stimuli. These models have broadly enabled the description of the angiogenic potential of macrophages under different stimulation scenarios by evaluating the activation and expression profiles of macrophage‐produced cytokines with known pro‐angiogenic properties (e.g., VEGF, EGF, FGF, MMPs) and also cellular transcription factors that are associated with angiogenesis (e.g., HIF1α, NFκB, AP‐1, STAT3; Hamik et al., 2006) .

Our group, through two modeling studies, has developed a novel large‐scale computational systems biology model formulated using mass‐action kinetics‐based ODEs to mechanistically characterize the complex biology of macrophage polarization spanning the M1–M2 spectrum and its potential impact on angiogenesis in health and disease (Zhao et al., 2019;  Zhao, Medeiros, et al., 2021). Calibrated and validated against over 250 sets of quantitative and qualitative experimental measurements (with over 800 datapoints) from literature and in‐house experiments, this model is the first in silico platform that can predictively simulate, from quantitative, temporal, dose‐dependent and single‐cell perspectives, the detailed dynamic expression and emergent activities of an array of macrophage phenotype markers (e.g., TNFα, IFNγ, interleukins, CXCLs, iNOS, ARG1, VEGF), essential signaling hubs (e.g., MAPKs, PI3K/AKT, IKK), cellular feedback regulators (e.g., microRNAs, SOCSs, A20), and key transcription factors (e.g., HIFs, NFκB, STATs, IRFs) ( Zhao, Medeiros, et al., 2021). Using the model, we created high‐resolution macrophage polarization maps that provide systems‐level information on the dynamic phenotype and overall function of macrophages in response to a diverse collection of polarizing conditions (Zhao & Popel, 2021). We further analyzed the model under hypoxia serum starvation (HSS), an in vitro condition for PAD, and revealed a distinctive macrophage phenotype including both M1 and M2 marker responses, which was also supported by in‐house experimental data under HSS at the level of individual markers (Ganta et al., 2019;   Zhao, Medeiros, et al., 2021). Then in the model sensitivity analysis, we pinpointed and evaluated the effectiveness of several targeted interventions that can potentially reprogram macrophages toward more pro‐angiogenic and anti‐inflammatory phenotypes under HSS.

### 3.3 Cancer cells

Within the tumor microenvironment, cancer cells themselves are a significant source of various cytokines and growth factors that can potently regulate cancer‐associated angiogenesis (Keeley et al., 2010). In the meantime, cancer cells constantly receive and process signals from the microenvironment, among which many are growth factors produced by autocrine and/or paracrine mechanisms (e.g., EGF, FGF, HGF, VEGF). To account for the highly complex biology and regulation in cancer cell signal transduction, Li and Mansmann constructed a first‐of‐its‐kind large‐scale multi‐pathway computational model that included over 30 ligand‐activated pathways and over 1000 functionally unique proteins to systematically describe the different hallmarks of cancer at the cell level (J. Li & Mansmann, 2013). Using this pan‐cancer model, the authors then analyzed the hallmark of angiogenesis (among other main model readouts), which is evaluated as a weighted score of the simulated profiles of pro‐ and anti‐angiogenic factors combined, under different scenarios of therapeutic interventions to predict individual‐specific treatment response. Later, Bouhaddou et al. and Frohlich et al. developed two new multi‐pathway computational models of cancer cell signaling, both with more detailed and mechanistic representations of the molecular interactions and smaller model scopes (each model includes about 100+ functionally unique gene/protein species; Bouhaddou et al., 2018; Frohlich et al., 2018). A significant step forward is that both models have successfully incorporated a large amount of quantitative data (e.g., time‐course trajectories, dose–response effects of different drugs, omics data from multiple cell lines) during model formulation and validation and therefore have achieved a higher level of overall predictive power for cell signaling and behavior. Of note, although these two models primarily focus on cancer cell apoptosis, their formulation also included several major angiogenesis‐related transcription factors (such as AP‐1, Myc, p53, FOXO), so that a systems‐level model‐based characterization of how cancer cells change their angiogenic potential under different conditions is feasible.

### 3.4 Fibroblasts

Fibroblasts, a major cell type in the stromal tissue of human organs, are primarily responsible for secreting molecules that make up the extracellular matrix (ECM). These cells also actively contribute to angiogenesis in normal physiology as well as in human diseases such as cancer and ischemic diseases (Koch et al., 2020; Meng et al., 2020; Newman et al., 2011; Sakamoto et al., 2020). Moreover, evidence has suggested that fibroblasts can dynamically respond to changes in the tissue microenvironment to critically reshape their angiogenic properties (Mouton et al., 2019). In order to achieve a systems‐level understanding of their intracellular biology, phenotype and function, Saucerman's group, through a series of modeling studies using logic‐based ODEs, has developed a unique systems biology modeling platform of fibroblast signaling and mechanistic phenotype regulation (Zeigler et al., 2016, 2020, 2021). With its primary focus on myocardial infarction (MI) and fibrosis, this in silico platform covers rich mechanistic details downstream of 10 different biochemical/mechanical stimuli‐driven pathways; it also includes a number of angiogenesis‐regulating transcription factors. With such an “in silico fibroblast” modeling platform, the authors then carried out various model‐based translational analyses, such as high‐resolution characterization of fibroblast phenotypes using MI animal data, as well as virtual drug target screening against cardiac fibrosis (Zeigler et al., 2016, 2020, 2021). Since this modeling platform is formulated with basic fibroblast signaling mechanisms, we therefore believe that it can also be smoothly tailored to comprehensively investigate how fibroblasts influences angiogenesis in MI and in other human diseases (e.g., PAD and cancer).

### 3.5 Cell–cell communication during angiogenesis

Endothelial cells communicate with other angiogenesis driver cells, including pericytes (Armulik et al., 2005), vascular smooth muscle cells (M. Li et al., 2018), macrophages (Baer et al., 2013), skeletal muscle cells (Haas et al., 2012; Olfert et al., 2016), and tumor cells in tumor angiogenesis (Lee et al., 2015; Lopes‐Bastos et al., 2016). Endothelial cells can communicate with other cells through a variety of mechanisms, including cell–cell adhesion, formation of junctional complexes, secretion of paracrine cytokines, and metabolite secretion. Below we discuss selected computational models that incorporate communication between endothelial cells and pericytes.

In capillaries and venules, pericytes wrap around endothelial cells to regulate vascular function. In addition to intracellular signaling pathways in the endothelial cells, the communications with pericytes also regulate vascular growth, inflammation, and homeostasis (Armulik et al., 2011). Kang et al. used a computational model validated by experimental studies of the interaction between pericytes and endothelial cells, demonstrating quantitatively how VEGF, TNF and pericytes modulate the pro‐ and anti‐inflammatory signaling via Notch/Jag1/Dll4 signaling to shift the endothelial cell fate (Kang et al., 2019). Walpole et al. developed an agent‐based model of retinal angiogenesis with endothelial–pericyte contact and intracellular signaling as mechanisms of cell fate determination to investigate the role of pericytes in the morphogenesis of microvascular network (Walpole et al., 2017).

In addition to VEGF/Notch signaling, endothelial cells and pericytes cross talk includes Ang/Tie, sphingosine‐1‐phosphate (S1P), PDGF, N‐cadherin, and transforming growth factor (TGF) pathways, as reviewed in Sweeney and Foldes (Sweeney & Foldes, 2018). Skeletal muscle angiogenesis involves exercise or hypoxia‐induced release of growth factors including VEGF and Ang, matricellular protein TSP‐1, and release of matrix‐metalloproteases (MMPs; Haas et al., 2012; Olfert et al., 2016). Endothelial‐macrophage interaction in angiogenesis includes growth factors, metabolites, and cytokines secreted by both endothelial cells and macrophages, including TNF, VEGF, and TGF, as reviewed in Baer et al. (2013). In tumor angiogenesis, endothelial cells communicate with cancer cells and fibroblasts though a variety of mechanisms, including the secretion of growth factors and cytokines by fibroblasts, the secretion of TGF and VEGF by cancer cells to promote endothelial migration and proliferation, integrin binding to CD151 to promote tumorigenicity, secretion of Slit2 and Perlecan by endothelial cells to suppress tumorigenicity, cleavage and release of soluble Jagged by ADAM17 to promote cancer stem cell differentiation, release of cytokines by endothelial cells to promote tumor invasion, and release of microRNAs that have anti‐angiogenic or pro‐angiogenic effects, as reviewed in Lee et al. and Choi and Moon (Choi & Moon, 2018; Lee et al., 2015). The cell–cell communication mechanisms between endothelial cells and pericytes, macrophages, fibroblasts, cancer cells, and skeletal myocytes are summarized in Figure 1.

Hutchinson et al. used a spatially averaged, ODE‐based multiscale model of the interaction between endothelial cells and pericytes with VEGF, ANG, and PDGF binding kinetics to demonstrate that the vascular phenotype changes can predict the response to combination treatments targeting VEGF, ANG2, or PDGF (Hutchinson et al., 2016). Jain and Jackson used a computational model of the interaction between endothelial cells and tumor cells to show that the bidirectional cell–cell communication between endothelial cells and tumor cells can explain the discrepancy in the effect of VEGF‐targeted therapies in vitro and in vivo (H. Jain & Jackson, 2018). Barnaby et al. further expanded the endothelial and tumor cell communication model and adapted it to prostate cancer to demonstrate that vascular leakage might explain the poor correlation of prostate‐specific antigen and tumor burden (Barnaby et al., 2021). These computational models that include cell–cell communications can help inform our understanding of the complex signaling network formed by different cell types during angiogenesis and capture behaviors that cannot be reproduced by isolated single cell‐type models.

## 4 MODELS OF BLOOD VESSEL SPROUTING AND FORMATION AT THE TISSUE LEVEL

Angiogenesis is inherently a multiscale physiological process that encompasses molecular interactions, cellular behaviors, and vascular formation at tissue and organ level. In the previous sections we summarized computational models at the subcellular and cellular scale. Sprouting angiogenesis, characterized by the formation of new blood vessels from existing ones, is orchestrated by a multitude of molecular and cellular processes, including the secretion of pro‐angiogenic factors such as VEGF and FGF by tissues in response to hypoxia or increased metabolic need, degradation of basement membranes by MMP, intra‐ and intercellular signaling‐mediated endothelial differentiation, migration, and proliferation, and vascular lumen formation (Ribatti & Crivellato, 2012). It should be noted that this review focuses primarily on sprouting angiogenesis, while other forms of angiogenesis, including intussusceptive angiogenesis, vascular co‐option and mimicry are important part of tumor vascularization and vascular remodeling (Ribatti & Pezzella, 2021). Computational models have been developed to facilitate our understanding of intussusceptive angiogenesis (Szczerba et al., 2009) and vascular co‐option (Ribatti & Pezzella, 2021), and integrative models describing multiple forms of angiogenesis during vascular remodeling have been reviewed in Rieger and Welter (2015). Here we discuss computational models that focus on sprouting angiogenesis and blood vessel formation at the tissue level as well as multiscale models that integrate molecular interactions at the molecular and cellular scale with tissue‐ and organ‐level models.

Computational models that simulate sprouting angiogenesis and cell migration are categorized into tip cell migration models, tip and stalk cell models, and cell shape dynamics models and reviewed in Heck et al. (2015). Qutub et al. summarized and focused on multiscale models of angiogenesis that integrate molecular and cellular processes with tissue‐ and organ system‐level models, and categorized them based on model methodology into discrete, continuous and hybrid models (Qutub et al., 2009). Logsdon et al. reviewed in detail the physiological events in the multistep process of sprouting angiogenesis and vessel remodeling, and the computational models that focus on different steps of the process (Logsdon et al., 2014). Below we focus on advances in tissue‐level and multiscale modeling of sprouting angiogenesis and vessel formation.

Using a multiscale systems biology model, Qutub and Popel simulated angiogenic sprouting with molecular and cellular level mechanisms, providing quantitative understanding of how the concentration distribution of VEGF and Delta‐like 4 Notch ligand direct angiogenic sprouting (Qutub & Popel, 2009). McDougall et al. used a hybrid PDE‐discrete model that tracks astrocyte and endothelial cell movement to simulate the development of retinal vasculature and blood perfusion (McDougall et al., 2012). Vega et al. used cellular pots model with Notch signaling dynamics to simulate tip cell selection and vessel branching to demonstrate that increased Jagged production can explain some features of the pathological vasculatures in cancer and diabetes (Vega et al., 2020). Moreia‐Soares et al. used both 2D and 3D computational models to demonstrate that the production of angiogenic factors by hypoxic cells promotes anastomosis during sprouting angiogenesis (Moreira‐Soares et al., 2018).

The agent‐based memAgent‐spring model developed by Bentley et al. (2008) has been incorporated in various computational models of sprouting angiogenesis to study different mechanisms affecting of VEGF/Notch and endothelial tip cell selection, including the regulation of VE‐Cadherin dynamics (Bentley et al., 2014), VEGF‐driven positive feedback through tetraspanin (Page et al., 2019), and active perception during angiogenesis through filopodia (Zakirov et al., 2021).

Norton and Popel developed a 3D agent‐based model framework that incorporates stalk cell proliferation and tip cell migration to quantitatively investigate their effect on vascular formation (Norton et al., 2018; Norton & Popel, 2016). The model can be used to inform experimental studies and drug development of combinations of anti‐angiogenic cancer therapies or pro‐angiogenic therapies in cardiovascular diseases and regenerative medicine that differentially target the migration and proliferation of endothelial cells.

In addition to angiogenesis, vascular remodeling and pruning also play important roles in the generation of microvascular network for efficient functioning of the microvasculature during angioadaptation (Pries & Secomb, 2014). Computational models that focus on the generation of microvascular networks and their transport properties during angioadaptation have been extensively reviewed in Pries and Secomb (2014) and Pries et al. (2010).

Walpole et al.'s model, briefly discussed in the previous section, used an agent‐based model with accurate representation of pericytes to demonstrate the role of pericytes in the signal propagation during retinal vascular formation (Walpole et al., 2017). Sugihara et al. developed a cellular Potts model‐based model of pericyte wrapping with mechanistically detailed PC‐ECM interaction (Sugihara et al., 2020). Stepanova et al. used a hybrid multiscale agent‐based model that incorporates VEGF/Notch signaling, endothelial migration dynamics, and tissue‐level ECM remodeling to quantitatively investigate cell mixing, the phenomenon in which cells dynamically rearrange and change the microenvironment (Stepanova et al., 2021). Nivlouei et al. developed a hybrid multiscale model that integrates: a Boolean model of signal transduction pathways involving receptor tyrosine kinases, integrin, cadherin, and Wnt; a cellular Potts model of interactions between cancer cell and endothelial cell in the ECM; and a PDE‐based diffusion model of VEGF and nutrients like oxygen (Jafari Nivlouei et al., 2021). The model was used to simulate tumor growth when one or several receptors are blocked. Computational models that focus on the role of MMPs have also been developed to obtain quantitative understanding of the invasion of angiogenic sprout into the ECM. Karagiannis and Popel used a computational model of MMP‐expressing angiogenic tip cell with detailed molecular mechanisms of protein interactions of MMPs and their inhibitors to quantitatively estimate the velocities of proteolytic tip cells (Karagiannis & Popel, 2006). Vempati et al.'s model focused on the role of MMP in releasing the ECM‐sequestered VEGF and quantitatively demonstrated that the cleavage of VEGF by ECM is likely to be mediated by multiple cells (Vempati et al., 2014).

## 5 MODELING THE EFFECT OF ANGIOGENESIS AND ANGIOGENESIS‐TARGETING DRUGS IN TISSUE/BODY LEVEL MODELS

Numerous drugs are developed to inhibit or induce angiogenesis in target tissues. The large number of potential targets and the lack of clinical response in some clinical trials highlight the need for computational tools to propose potent targets and predictive biomarkers to accompany the clinical effort in development of anti‐/pro‐angiogenic treatments (Teleanu et al., 2019). Computational models, particularly those at tissue‐ and whole‐body scales, provide an effective tool to predict disease progression upon administration of drugs of interest. In this section, we expand the topic from tissue‐level to whole‐body level modeling of angiogenesis. Whereas a number of models were built to visualize heterogeneous spatiotemporal vascular morphology in diseases based on advanced imaging techniques (Nikmaneshi et al., 2020; Phillips et al., 2020; Stamatelos et al., 2014, 2019; Suzuki et al., 2018), we focus on models that derived therapeutic implications for complex diseases, such as cancer and PAD. The incorporated angiogenic pathways and highlighted therapeutic implications derived from the reviewed models are summarized in Table 2.

**TABLE 2:** Computational models of angiogenesis on tissue‐ and whole‐body levels with therapeutic implications

Reference	Angiogenic pathways	Disease	Model type	Therapeutic implications
Tissue level				
(Liao et al., 2014)	Secretion of VEGF under mild hypoxic conditions regulated by IL‐35 and oxygen level Endothelial cell recruitment and proliferation facilitated by VEGF Oxygen delivery by endothelial cell, diffusion, and cellular consumption Oxygen‐dependent cancer cell proliferation and necrosis.	Plasmacytoma cells (J558)‐injected mice models (with normal or high IL‐35 secretion)	Partial differential equation (PDE)	Tumors with higher VEGF and IL‐35 production have stronger response to anti‐IL‐35 treatment
(Lai et al., 2018)	VEGF secretion by cancer cell and M2‐type macrophage Endothelial cell recruitment and proliferation facilitated by VEGF Oxygen delivery by endothelial cell, diffusion, and cellular consumption Oxygen‐dependent cancer cell proliferation.	Murine breast tumor model	PDE	Bromo‐ and Extra‐Terminal (BET) protein inhibitor, which suppresses TNF‐α secretion by M1‐type macrophage, cancer cell proliferation, and VEGF‐A secretion, improves antitumor response when combined with anti‐CTLA‐4 treatment
(Vavourakis et al., 2018)	Biochemical solver module (dynamic of tumor‐angiogenic factors, oxygen, and ECM degrading enzymes) Solid solver module (tumor growth and structural integrity of ECM) Vascular network module (capillary sprouting, wall remodeling, etc.) Fluid solver module (interstitial, intra‐ and trans‐vascular flow rates, pressure differences, etc)	Solid tumors in murine models	PDE, rule‐based algorithm	High tumor vascular pore size and cytotoxic drug binding affinity to target enhance drug delivery into the tumor interstitial space via diffusion and tumor regression. Cytotoxic drug delivery normalizes tumor vasculature, reducing tissue hydrostatic and interstitial fluid pressures
(Liang et al., 2019)	Cell‐cycle, EGF and VEGF receptor pathways (molecular scale) Phenotypic changes of cancer cell and endothelial cell (cellular scale) Growth factors, nutrients, and drug transport (microenvironment scale) Blood vessel growth and branching (tissue scale).	Murine brain tumor model	Ordinary differential equation (ODE), rule‐based algorithm, PDE	Cancer cell survival rate decreases upon inhibition of EGF receptor and rebounds due to drug resistance, which can be reversed by inhibition of VEGF receptor. Inhibition of both VEGF and EGF receptors shows synergistic effect
(Mpekris et al., 2017, 2020)	Tumor oxygenation by functional vasculature, which facilitate cell proliferation and immune activation Functional vascular density regulated by blood vessel compression and IFNγ concentration Blood vessel compression by solid stress level, cancer and endothelial cell densities Solid stress level increased by tumor stroma components, which is mediated by CXCR4/CXCL12 signaling Ang1 and Ang2, PDGF‐B, VEGF, and CXCL12 secretion induced by hypoxia, which regulates stabilization and migration of endothelial cell.	Generic murine tumor models	PDE	Vascular normalization via low doses of anti‐VEGF treatment, when combined with immunotherapy sequentially, leads to optimal treatment efficacy Stroma normalization via targeting of CXCL12/CXCR4 signaling further enhance the efficacy of immunotherapy by improving functional vascular density, which has additive effect with anti‐VEGF treatment
(Perfahl et al., 2020)	Cell‐cycle pathways with VEGF release (subcellular scale) Cell migration and phenotypic change (cellular scale) VEGF and oxygen distribution (diffusible scale) Vessel sprouting and migration and pressure calculation (vascular scale).	Hepatocellular carcinoma in human	ODE, rule‐based algorithm, PDE	When trans‐arterial chemoembolization therapy eradicates all or majority of the tumor vasculature, it creates a short therapeutic window for oxygen enhancement therapy to inhibit hypoxia‐induced VEGF secretion and thus prevent tumor revascularization, improving long‐term clinical response
Whole‐body level				
(Clegg et al., 2017; Clegg & Mac Gabhann, 2017, 2018)	Secretion and distribution of VEGF_165_, VEGF_121_, VEGF_189_, PlGF1, PlGF2, sR1 in blood, main body tissues, and the calf muscle Dynamics of VEGFR1, VEGFR2, and NRP1 on endothelial cell Interactions among VEGFs, PlGFs, their corresponding receptors, and extracellular GAGs Phosphorylation of VEGFR2 Dynamics of VEGF_165a_ and VEGF_165b_	Peripheral arterial disease in human	ODE	VEGF_165b_ is not a good biomarker or target for pro‐angiogenic therapy Sub‐saturating VEGFR2 activation is critical to sustain therapeutic angiogenesis via biomaterial‐based VEGF delivery Inducing alternative splicing from VEGF_165b_ to VEGF_165a_ via gene therapy shows promising effect Antibody therapy targeting all VEGF isoforms increases free VEGF_165a_ level and VEGFR2 activation in target tissue, while anti‐VEGF_165b_ therapy has minimal effect
(Gaddy et al., 2017; Q. Wu et al., 2018)	Secretion of human VEGF isoforms in tumor (i.e., VEGF_121_, VEGF_165_) Secretion of mouse VEGF isoforms in blood and peripheral tissues (i.e., VEGF_120_, VEGF_164_) Expression of VEGF receptors (VEGFR1, VEGFR2) and co‐receptors (NRP1, NRP2) Dynamics of VEGF isoforms and their receptors in blood, tumor, and normal tissues VEGF‐mediated tumor growth	Murine model with breast tumor xenograft	ODE	Low levels of VEGF receptor and high tumor NRP levels are predicted to increase the efficacy of anti‐VEGF treatment Linear growth rate of tumor and its ratio to exponential growth rate are identified to be prognostic biomarkers of the population survival outcome
(D. Li & Finley, 2018)	Secretion of active VEGF isoforms (i.e., VEGF_121_, VEGF_165_) and the inactive VEGF isoform, VEGF_114_ Expression of VEGF receptors (VEGFR1, VEGFR2) and co‐receptors (NRP1, NRP2) Interactions between TSP1 and its receptors (i.e., LRP1, CD36, CD47, α_x_β_1_ integrins) VEGF cleavage by MMP3, proMMP9, and MMP9 regulated by TSP1 GAG binding with TSP1 and VEGF_165_ in the interstitial space α2 M binding with VEGF in the blood	Breast cancer in human	ODE	Combination treatment using bevacizumab and a peptide mimetic of TSP1 shows additive effect in shifting tumor angiogenic balance Patients with low levels of VEGFR1 or CD47 and/or high level of CD36 on tumor cell are predicted to have strong clinical response to the combination therapy
(H. Wang et al., 2021)	Modified Gompertzian tumor growth dynamic Secretion of tumor angiogenic factor Effect of cytotoxic drugs on tumor angiogenic factor and endothelial cell.	Triple‐negative breast cancer in human	ODE	Densities of CD8^+^ and CD4^+^ T cell in tumor are identified as predictive biomarkers for combination therapy of PD‐L1 inhibition and nab‐paclitaxel. Concurrent treatment of PD‐L1 inhibitor with metronomic nab‐paclitaxel dosing have greater tumor size reduction comparing to sequential treatments

Abbreviations: α2M, alpha‐2‐macroglobulin; Ang, angiopoietin; CTLA‐4, cytotoxic T‐lymphocyte‐associated protein 4; ECM, extracellular matrix; EGF, epidermal growth factor; GAG, glycosaminoglycans; IFNγ, type II interferon; MDSC, myeloid‐derived suppressor cell; MMP, matrix metalloproteinases; NRP, neuropilin; PD‐(L)1, programmed death‐1 (ligand); PDGF, platelet‐derived growth factor; PlGF, placental growth factor; sR1, soluble VEGF receptor; TNF, tumor necrosis factor; TSP, thrombospondin; VEGF, vascular endothelial growth factor.

Chemotherapy, which has been used as a standard‐of‐care treatment for multiple cancer types for decades, is known to have anti‐angiogenic effects in addition to its cytotoxic effect on cancer cells (Kerbel & Kamen, 2004). However, the mechanisms of action through which chemotherapy inhibits tumor angiogenesis are not always fully understood due to the complexity of tumor vascularization, which involves dynamics at multiple scales. Particularly, metronomic chemotherapy, in which low doses of cytotoxic drug are administered on a frequent schedule, showed stronger anti‐angiogenic activity than maximum‐tolerated dose regimens (Kerbel & Kamen, 2004). To investigate the dominant mechanism during metronomic dosing of chemotherapy, Mpekris et al. incorporated hypothesized mechanisms of metronomic chemotherapy into a PDE‐based model, focusing on TSP‐1‐induced vascular normalization (Mpekris et al., 2017). The model predicted that vascular normalization is a prerequisite of stronger antitumor effect observed in metronomic chemotherapy compared to maximum‐tolerated dosing and was validated against results from three in vivo experimental studies (Mpekris et al., 2017). Additionally, multiscale models were built to simulate tumor response to chemotherapy in mice and humans with an emphasis on changes in tumor vasculature during the treatment (Perfahl et al., 2020; Vavourakis et al., 2018). These models together provide valuable insights into how chemotherapy can affect tumor vascularization from molecular scale to tissue scale and how treatment outcomes can be optimized via follow‐up treatments.

Besides chemotherapy, many drugs are developed to directly target pro‐angiogenic receptors and inhibit tumor vascularization in patients with cancer. Although anti‐angiogenic treatments prevent transport of oxygen and nutrients into the tumor, which inhibits cancer cell survival rate, angiogenesis and other resistance mechanisms can be promoted in the hypoxic environment created by prolonged anti‐angiogenic treatment and eventually lead to tumor relapse and metastasis (R. K. Jain, 2014). Therefore, dose optimization is critical in anti‐angiogenic treatment, which is difficult to investigate in clinical settings. Based on experimental results that IL‐35 secretion by tumor cells facilitates angiogenesis in plasmacytoma cells (J558)‐injected mice, Liao et al. developed a PDE‐based model that integrates interactions between immune cells and tumor cells, which are mediated and regulated by cytokines and oxygen delivery by endothelial cells (Liao et al., 2014). The model was used to conduct in silico experiments with similar settings to an in vivo experimental study, generating predictions of myeloid cell infiltration, VEGF dynamics, and FoxP3+/CD8+ ratio that are consistent with the reported in vivo measurements (Z. Wang et al., 2013). The model further predicted that continuous anti‐IL‐35 treatment would have a better anti‐angiogenic effect than intermittent dosing (Liao et al., 2014). From the same group, Lai et al. predicted that the efficacy of bromo‐ and extra‐terminal (BET) protein inhibitors, when combined with anti‐CTLA‐4 treatment, increases as the dose of each drug increases (Lai et al., 2018). This prediction was later confirmed by an in vivo study on murine prostate cancer model, where an additive effect between BET inhibition and anti‐CTLA‐4 treatment was observed (Mao et al., 2019). Based on these findings, the optimal regimen can be determined to balance drug efficacy with adverse effects, which is also positively correlated with the drug doses.

Using a hybrid approach, Liang et al. simulated tumor vascular growth upon inhibition of EGFR in mice with brain tumors and observed a rebound of cancer cell survival rate after a short period of antitumor response (Liang et al., 2019). By applying anti‐VEGF treatment at various time points during inhibition of EGFR, they concluded that angiogenesis is the dominant mechanism leading to resistance to EGFR inhibitor and that combination with anti‐VEGF treatment is able to inhibit tumor relapse, which was validated by in vivo experimental data from mice with brain tumors (Tonra et al., 2006). Similarly, by incorporating therapeutical effect of various drugs of interest (e.g., anti‐angiogenic agent, CXCR4 inhibitor, anti‐PD‐1 and anti‐CTLA‐4 antibodies), Mpekris et al. built a hybrid model upon their previous studies (Mpekris et al., 2020). Validated against data from five in vivo studies mostly on breast tumor, the model was used to visualize spatial distributions of tumor stromal and vascular components during mono‐ and combination therapies and predicted a therapeutic window for optimal treatment efficacy for combination of anti‐VEGF and immunotherapies.

**FIGURE 2:**
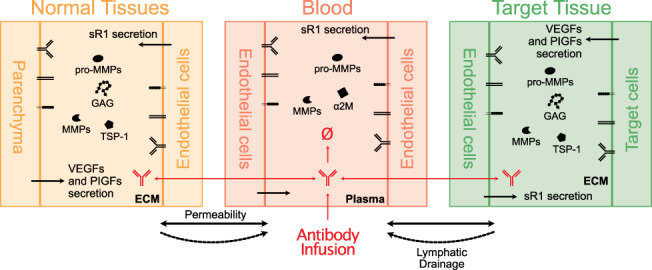
Whole‐body compartmental model structure. Isoforms of VEGF and PlGF are secreted by parenchymal and target cells (e.g., cancer cell, calf muscle cell in PAD, etc.), which can bind to GAG, TSP‐1, α2M, receptors on cellular surface, and sVEGFR1 (sFlt1) secreted by endothelial cell. Angiogenic pathways can also be regulated by matrix metalloproteinases (e.g., MMP2, MMP3, MMP9). Ligands and sVEGFR1 can be transported between the blood and the interstitium of tissues via vascular permeability and/or lymphatic drainage

In addition to PDE‐based models, which retain spatial heterogeneities in the tumor microenvironment, ODE‐based models are developed on the tissue‐level as well as on the whole‐body scale to incorporate more mechanistic details and therapeutic implications not only in the target tissue, but also in the blood and normal tissues (Figure 2; Clegg & Mac Gabhann, 2017, 2018; Duda et al., 2013; Finley et al., 2015; Finley & Popel, 2013; Gaddy et al., 2017; D. Li & Finley, 2018; H. Wang et al., 2021; F. T. Wu et al., 2010b; Q. Wu et al., 2018; Zhao, Heuslein, et al., 2022 ). The assumption of spatially averaged cellular density and molecular concentration allows ODE‐based models to maintain a moderate computational cost with increased complexity, and faithfully recapitulate the drug pharmacokinetics and pharmacodynamics (PK/PD). Unlike the PDE‐based models above, in which drug effect is achieved by manipulating target‐related parameters, whole‐body level models can better capture the interindividual variabilities in pharmacodynamics by considering variations in the blood and nontarget tissues, which also play critical roles in drug efficacy and toxicity (Turner et al., 2015). By incorporating interactions between all known isoforms of VEGF and PlGF and their corresponding receptors in the blood and peripheral tissues, Clegg et al. developed an ODE‐based systems pharmacology model, which was validated by comparing the model‐simulated anti‐VEGF165b treatment with murine experimental data (Clegg & Mac Gabhann, 2018). Interestingly, the model predicted that anti‐angiogenic treatment with a non‐isoform‐specific VEGF antibody will promote VEGFR2 phosphorylation in target tissue with PAD. This finding may seem counterintuitive but can be explained by the “shuttling” effect of anti‐VEGF antibody (Clegg & Mac Gabhann, 2018; Finley & Popel, 2013). Similar models are also developed to predict antitumor effects of the anti‐angiogenic treatment in mice and humans with breast cancer (Gaddy et al., 2017; D. Li & Finley, 2018; H. Wang et al., 2021; Q. Wu et al., 2018). Importantly, by combining the pharmacokinetic modeling of therapeutic agents, whole‐body level models can provide valuable therapeutic implications when investigating the systemic effect of anti‐/pro‐angiogenic therapies.

## 6 OMICS DATA‐DRIVEN SYSTEMS BIOLOGY STUDIES RELATED TO ANGIOGENESIS

Omics refers collectively to genomics, proteomics, transcriptomics, and metabolomics (Hasin et al., 2017). With increasing applications to different life sciences fields, omics sciences have provided means for identifying, quantifying, and characterizing the most influential biological molecules and structures in cells, tissues, and organisms. Systems biology approaches have been often integrated with omics, sometimes multi‐omics, to study diseases, treatments, and behaviors through the discovery of pathway‐based biomarkers, development of global gene/protein interaction maps, identification of essential driver genes in disease, which together provide useful holistic frameworks to advance the understanding of complex biological processes in human diseases (Oulas et al., 2019). In tumor studies, for example, Munaron presented a review of systems biology approaches used for analyzing ion channels and transporters in tumor angiogenesis, focusing on the endothelial transportome diversity (Munaron, 2015). Additionally, this integrative concept has been used for identifying drug modulation of angiogenesis‐related pathways, such as performed in an investigation of an anti‐angiogenic drug (Aflibercept) through analyzing protein–protein interaction networks and multi‐experiment microarray data (Latifi‐Navid et al., 2021). This section reviews the omics literature applied to systems biology methods with a focus on angiogenesis in different disease areas (tumor, wound healing, cardiovascular, and ocular diseases). Table 3 summarizes the studies pertinent to this review.

**TABLE 3:** Computational omics, data‐driven systems biology studies in angiogenesis

Reference	Disease	Omics	Machine learning applied	Results related to angiogenesis	Goal	Enrichment analysis/interaction network
Tumor						
(Zheng et al., 2021)	Kidney renal clear cell carcinoma	Genomics Transcriptomics	Yes	Feature genes correlated to angiogenesis were found (MMRN2, CLEC14A, ACVRL1, EFNB2, TEK). The top three transcription factors regulating angiogenesis found were RFX2, SOX13, THRA	Diagnosis and treatment	Gene set enrichment/transcription factor enrichment
(J. T. Chen et al., 2018)	Colon cancer	Proteomics Secretomics Transcriptomics Translatomics	No	Secretory proteins IGFBP6 and LOXL2 (hypoxia biomarkers) were upregulated in hypoxic HCT116 cells. Hypoxia is correlated with increased risk of tumor invasion and metastasis	Diagnosis and treatment	Gene ontology enrichment/protein–protein network
(Motzer et al., 2020)	Renal cancer	Transcriptomics Genomics	No	Somatic mutations in PBRM1 and KDM5C associate with high angiogenesis. Sarcomatoid tumors exhibited a lower prevalence of PBRM1 mutations and angiogenesis markers	Treatment	Pathway enrichment
(Goveia et al., 2020)	Lung cancer	Transcriptomics Genomics Proteomics	Yes	Collagen modification identified as a potential angiogenic pathway	Treatment	Gene set enrichment
(Hoang et al., 2019)	Lung cancer	Metabolomics Genomics Transiptomics	No	Metabolites and genes that contribute to angiogenesis and cell proliferation were found to be predominant in both subtypes of lung cancer. ADP was pointed as a potential therapeutic target for NSCLC	Treatment	Pathway enrichment/metabolite set enrichment
(Lin et al., 2020)	Bladder urothelial carcinoma	Transcriptomics Radiomics	Yes	Radiomics signature developed might reflect angiogenesis status of BLCA patients	Survival	Functional enrichment
(Lucarelli et al., 2018)	Clear cell–renal cell carcinoma (ccRCC)	Metabolomics Genomics Transcriptomics	Yes	NDUFA4L2 found as the most highly expressed gene in renal cancer cells and possesses a role in angiogenesis	Diagnosis and treatment	Metabolite set enrichment/gene set enrichment/biochemical pathway enrichment
(D'Arcangelo et al., 2016)	Melanoma	Transcriptomics miRNomics	No	miR‐424 has been implicated in angiogenesis regulation	Treatment	Gene set enrichment
Wound healing						
(Beyer et al., 2018)	Wound	Sphingolipidomics Proteomics Secretomics	No	M2 secreted factors FGF‐2 and VEGF were present in M1 media. Angiogenesis onset was initiated by a decline of inflammation‐associated factors	Treatment	N/A
(Mahmoudi et al., 2019)	Wound	Transcriptomics Metabolomics Epigenomics Secretomics	Yes	Old fibroblast secreted cytokines (e.g., IL‐6 and TNF) that induced inflammatory signaling pathways modulating the reprogramming efficiency of iPS cells	Treatment	Pathway enrichment
Cardiovascular disease						
(Fu et al., 2020)	Peripheral arterial disease	Transcriptomics Proteomics	No	Hypoxia‐induced IL‐35 inhibited hindlimb ischemia inflammatory angiogenesis at an early phase, but spared regenerative angiogenesis at a late phase	Treatment	Gene set enrichment
(Chu, Vijay, et al., 2015)	Peripheral arterial disease	Angiomics Immunomics Arteriomics	Yes	TLR4,THBS1, PRKAA2, EphA4, TSPAN7, SLC22A4, and EIF2a were identified as potential targets for treatment	Treatment	Gene set enrichment/protein–protein interaction networks
(Figliolini et al., 2020)	Peripheral arterial disease	Transcriptomics Proteomics	No	Transcriptomics showed adipose stem cell‐derived extracellular vesicles have several pro‐angiogenic mRNAs (fibronectin 1, MMP2, angiopoietin, FGF2, VEGFA, and FLT1 mRNA)	Treatment	Pathway enrichment
(Potz et al., 2017)	Ischemic myocardium	Proteomics	No	Calpain inhibition was found to promote angiogenesis in the myocardium by upregulating VEGF receptors 1 and 2, VE‐Cadherin, pVE‐cadherin, y‐catenin, and B‐catenin. It also increased the expression of total GSK‐3B	Treatment	N/A
(Pepin et al., 2021)	Diabetic vasculopathy	Genomics Epigenomics Transcriptomics	No	Differential glucose‐dependent methylation and gene expression of VEGF and NOS3 were found	Diagnosis	Pathway enrichment
Ocular disease						
(Emri et al., 2020)	Age‐related macular degeneration	Transcriptomics Proteomics Secretomics	No	Zinc supplementation affects molecular pathways important for angiogenesis, such as that of TGFB	Treatment	Pathway enrichment
(Chai et al., 2020)	Orbital venous malformations	Transcriptomics	No	Epidermal growth factor (EGF) and Leptin are upregulated in OVM patients	Diagnosis	Pathway enrichment
(J. Zhang et al., 2020)	Diabetic retinopathy	Genomics	No	Angiogenesis‐associated genes were inferred (e.g., CXCR2, IL11, CSF3, FGF23, VEGFD)	Diagnosis	Pathway enrichment/protein–protein interaction network
(Huang et al., 2020)	Diabetic retinopathy and diabetic macular edema	Transcriptomics	No	TGFB1I1 and TGFBR3 gene expressions were upregulated in the TGF‐Beta signaling pathway	Diagnosis and treatment	Pathway enrichment/gene–gene interaction network
(Jo et al., 2016)	Induced retinal vascular hyperpermeability	Proteomics	No	β2 integrin was identified as a potential therapeutic target against VEGF‐induced vascular hyperpermeability	Treatment	Pathway enrichment/protein–protein interaction network
(Shao et al., 2019)	Diabetic retinopathy	Transcriptomics	No	BTG1, ABL1, RRAS, ITGA5, JAK1, ANXA3, HMOX1 were found as angiogenesis regulating genes for mRNAs regulated by glucose and TTR	Diagnosis and treatment	Pathway enrichment/gene set enrichment/protein–protein interaction networks
(Saddala et al., 2020)	Diabetic retinopathy	Proteomics Transcriptomics	No	Downstream targets of PlGF with a role in human retinal endothelial cells were found, such as PRDX6, HMOX1, NQO1, and YES1	Treatment	Pathway enrichment/protein–protein interaction network
(Dong et al., 2021)	Diabetic retinopathy	Transcriptomics	No	BMP4 and SMAD9 genes acted on angiogenesis in DR	Treatment	Pathway enrichment
(Yan et al., 2021)	Diabetic retinopathy	Proteomics	No	Melatonin alleviated dysfunction and inflammatory activation in DR, inhibiting Wnt/Beta‐catenin pathway, leading to attenuation of angiogenesis and iBRB disruption	Treatment	Pathway enrichment/protein–protein interaction networks

### 6.1 Omics data‐driven systems biology studies of tumor angiogenesis

Tumor vascularization is an established factor in tumor growth and progression, either through inducing new blood vessel formation or through alternative vascularization patterns such as co‐option of preexisting blood channels, intussusceptive angiogenesis and vascular mimicry (Kuczynski et al., 2019; Ribatti & Pezzella, 2021). Network bioinformatics approaches have been important for advancing tumor angiogenesis research in terms of providing quantitative mechanistic insights from molecular and pathway levels and aiding experimental designs (Rivera et al., 2012). D'Arcangelo et al. assessed melanoma using a multi‐omics approach (D'Arcangelo et al., 2016); based on transcriptomics and miRNAomics, the authors were able to find several clues on the pathways affected by PDGFRα, a known inhibitor of angiogenesis and melanoma proliferation. Zheng et al. applied genomic and transcriptomic analyses to find new potential targets for prognosis and treatment for patients with clear cell renal cell carcinoma (ccRCC), using a novel angiogenesis score calculated based on the expression of over 180 genes (Zheng et al., 2021). Lucarelli et al. used integrative omics analysis of ccRCC involving metabolomics, genomics, and transcriptomics and identified NDUFA4L2 (NADH dehydrogenase 1 alpha subcomplex 4‐like 2) as the gene most highly expressed in ccRCC, and further they experimentally showed that this gene can functionally participate in cancer cell‐mediated angiogenesis as well as several other biological processes that affect cancer growth (Lucarelli et al., 2018). In terms of informing biomarker selection for anti‐angiogenic therapies, Motzer et al. applied multi‐omics (transcriptomics and genomics) to the analysis of 702 pretreatment renal cell carcinoma tumor samples and identified disease molecular subtypes that are strongly associated with differential clinical outcomes to combined anti‐VEGF (bevacizumab) plus anti‐PDL1 (atezolizumab) versus sunitinib alone (Motzer et al., 2020).

In lung cancer, using single‐cell RNA data combined with bulk multi‐omics, Goveia et al. developed a detailed molecular atlas of tumor endothelial cell phenotypes as a means to better characterize endothelial cell heterogeneity in tumor and identify new targets for anti‐angiogenic strategies (Goveia et al., 2020). Hoang et al. combined metabolomic, transcriptomic, and genomic analyses to investigate differences in the metabolic profiles of two subtypes of non‐small‐cell lung cancer (lung squamous cell carcinoma versus lung adenocarcinoma) and identified adenosine diphosphate as a key metabolite that may critically contribute to angiogenesis and metastasis in lung cancer (Hoang et al., 2019). In colon cancer cells, an integrative multi‐omics analysis (combining proteome, secretome, transcriptome, translatome) has been applied to the identification of hypoxia‐regulated genes (including several genes that may notably regulate angiogenesis; J. T. Chen et al., 2018). In bladder urothelial carcinoma (BLCA), Lin et al. developed data‐driven risk models to predict patient survival based on radiomics, transcriptomics, and clinicopathological data, finding a newly defined radiomics signature as a potential indicator of tumor angiogenesis status in BLCA patients (Lin et al., 2020).

### 6.2 Omics data‐driven systems biology studies of angiogenesis in wound healing

In a wound microenvironment, angiogenesis performs an important role in tissue regeneration, leading to the development of capillary sprouts in the ECM through endothelial cell proliferation and forming a network of structures that can expand and branch. Following an injury, factors that stimulate angiogenesis become dominant, activating the angiogenic process and initiating tissue healing (Honnegowda et al., 2015). Several dynamical computational models of angiogenesis in wound healing have been developed, bringing insight regarding therapeutic targets and intervention strategies for restoring angiogenesis (Broszczak et al., 2017; Flegg et al., 2015; Nagaraja et al., 2019); however, there is still limited knowledge on the molecular and cellular mechanisms in physiological versus impaired wound healing (Stolzenburg‐Veeser & Golubnitschaja, 2018). Beyer et al. examined the protein secretome and sphingolipidomics data from differentially polarized macrophages and developed an in vitro model that can physiologically represent the endothelial‐macrophage cross talk to better dissect the roles of inflammatory factors on angiogenesis during wound healing (Beyer et al., 2018). Mahmoudi et al. performed multi‐omics profiling (transcriptomics, metabolomics, epigenomics) of fibroblasts from young and old mice and investigated the reprogramming efficiencies of such fibroblast‐derived induced pluripotent stem cells in vitro and in vivo to better understand how wound healing can be influenced by aging‐related cellular heterogeneity; this omics‐based experimental protocol may be further adapted to probe into the key angiogenesis‐related signatures that potentially drive the age‐related differences in wound healing (Mahmoudi et al., 2019).

### 6.3 Omics data‐driven systems biology studies of angiogenesis in peripheral arterial disease

Studies in the cardiovascular field have also started to integrate multi‐omics data (Alexandar et al., 2015; Leon‐Mimila et al., 2019; Pepin et al., 2021; Potz et al., 2017; Vilne & Schunkert, 2018). Peripheral arterial disease (PAD) is a cardiovascular disease that has a very high prevalence worldwide (Virani et al., 2021), and it is marked by narrowing or blockage of arteries in the lower limb usually due to atherosclerosis (plaque build‐up inside the vessel). A treatment strategy that has been commonly assessed for PAD is therapeutic angiogenesis (Annex & Cooke, 2021). Below we discuss several examples that have utilized such omics‐based systems biology approaches in the study of PAD pathophysiology.

With a particular focus on angiogenesis, Chu et al. in a foundational study constructed the angiome, a protein–protein interaction network (PIN) built from 11 public data sources and microarray datasets for identifying essential genes associated with angiogenesis in different disease settings (Chu et al., 2012). An application of the angiome network is presented in a subsequent study, in which the network is combined with gene expression datasets from three endothelial cell lines treated with VEGF to investigate the resulting different activation patterns of an array of tyrosine kinase receptors and their downstream signaling (Chu et al., 2014). Further in the context of PAD, Chu et al. expanded the angiome framework by developing PADPIN, which refers to protein–protein interaction networks of angiogenesis, arteriogenesis, and inflammation in PAD (Chu, Vijay, et al., 2015). By combining PADPIN with gene expression data collected from ischemic and nonischemic gastrocnemius muscles from two mouse strains (C57BL/6 and BALB/c) subjected to hindlimb ischemia (a commonly used experimental PAD mouse model), the authors identified a list of genes with potential therapeutic significance in PAD and demonstrated the feasibility of using such an omics‐based in silico platform for PAD research. Later, PADPIN was also combined with extensive drug‐target relationships derived from public drug databases to facilitate the identification of potential drug repositioning candidates for PAD from the aspect of promoting angiogenesis and alleviating inflammation (Chu, Annex, & Popel, 2015).

Fu et al. characterized the overall effect of IL‐35 in different mouse models (gain‐of‐function and loss‐of‐function), and by systematically analyzing omics data (proteomics and transcriptomics) they were able to show mechanistically that IL‐35 can potently impact ECM remodeling through collagen formation and integrin activation as well as ROS generation in endothelial cells to collectively suppress angiogenesis in the ischemic tissue (Fu et al., 2020). Also using the hindlimb ischemia model, Figliolini et al. showed the protective potential of adipose stem cell‐derived extracellular vesicles (ASC‐EVs) in post‐ischemia tissue neovascularization and regeneration; by performing transcriptomic and proteomic analyses, the authors further demonstrated that the protective effect is attributed to various pro‐angiogenic mRNA cargo and a key protein, neuregulin 1, in those ASC‐EVs (Figliolini et al., 2020).

### 6.4 Omics data‐driven systems biology studies of angiogenesis in ocular diseases

The ocular environment is subject to the formation of new blood vessels from existing capillaries in the eyes (ocular neovascularization) during normal development and repair. However, under inflammatory conditions, hypoxia, or aberrant development, ocular neovascularization also takes place, becoming the cause for several ocular diseases, such as diabetic retinopathy, age‐related macular degeneration, retinal vessel occlusion, and retinopathy of prematurity (Campochiaro, 2013, 2015). These diseases, along with orbital venous malformations and induced retinal vascular hyperpermeability have been assessed in several omics‐based studies during the past years (Chai et al., 2020; Dong et al., 2021; Emri et al., 2020; Jo et al., 2016).

A systematic analysis based on protein–protein interaction (PPI) network of angiogenesis‐related proteins suggested that the therapeutic regulation of multiple targets instead of a single factor like VEGF might be more effective in the treatment of pathological ocular neovascularization (Cabral et al., 2017). Additionally, a number of computational studies have employed network algorithms such as random walk with restart, shortest path and Laplacian heat diffusion to evaluated PPI networks in the STRING database to infer genes associated with the pathogenesis of proliferative diabetic retinopathy (DR; J. Zhang et al., 2020). From the omics perspective, high‐throughput approaches (e.g., ligandomics) have been used in the identification of novel and disease‐specific drug targets that control angiogenesis and vascular leakage in ocular diseases (LeBlanc et al., 2015; Rong et al., 2019). In addition, by analyzing quantitative proteomic data, Yan et al. elucidated the protective effects of melatonin in DR by showing that it inhibits the Wnt/β‐catenin pathway to reduce disease‐related angiogenesis, autophagic dysfunction, and inflammatory activation (Yan et al., 2021). In the work by Shao et al., transcriptomic analysis was used to identify long noncoding RNAs (lncRNAs) involved in the protective mechanisms of transthyretin (TTR), which is capable of repressing neovascularization in DR, and to examine the potential of these lncRNAs as diagnostic biomarkers (Shao et al., 2019). In non‐proliferative DR specifically, researchers have utilized transcriptomic and proteomic analyses to assess changes in the physiology of human retinal endothelial cells in the presence or absence of placental growth factor (PlGF) signaling and pinpointed the pentose phosphate pathway and TGFβ pathway as key mechanistic components in PlGF‐induced endothelial barrier dysfunction in DR (Huang et al., 2020; Saddala et al., 2020). A more complete summary of omics‐based studies of pathological ocular angiogenesis can be found in Table 3.

The review presented in this section shows the current state of omics data used in systems biology studies, with promising results presented for research on different diseases. Computational models describing physiopathological events are limited to biological data availability, and technological improvements in the past years have led to an increase in the availability of omics data, which provide means to developing better models for studying diseases such as the ones listed in this section. Despite the increase in computational burden and complexity that comes from adding new data to a model, such addition improves its accuracy, validity, and generalizability. Implementing machine learning or deep learning algorithms to interpret, analyze and implement omics data and model structure is a way of improving omics‐based computational models.

## 7 CONCLUSION

By nature, angiogenesis occurs as an outcome of complex signaling. First, pro‐ and anti‐angiogenic mediators are differentially produced and secreted as a result of the different intracellular signal transduction programs in different cell types, driven by environmental cues such as hypoxia and biochemical stimuli from cell–cell communications. Second, endothelial cells, as the predominant effectors, dynamically compute cell‐fate decisions based on a multitude of signals to accordingly form the new blood vessels, together with other cells. The complexity further builds up as these two sets of activities happen in parallel and in fully interactive and continuous manners. As reviewed here, systems biology models have greatly advanced our ability to probe into and interpret this complexity, from the perspectives of elucidating single‐/multi‐pathway intracellular networks, characterizing tissue‐level vessel sprouting and formation, simulating whole‐body distribution kinetics of angiogenesis biomarkers, and mapping and uncovering novel multilevel features of pathophysiological angiogenesis from multi‐omics. However, as more mechanistic details are incorporated into complex systems biology models, dealing with a large number of parameters is a general challenge during model calibration and validation. For that, quantitative methods such as sensitivity analysis and uncertainty quantification have proved essential for analyzing model robustness (Marino et al., 2008; Musuamba et al., 2021), In addition, despite that identifiability and complexity issues are common for sophisticated models, sensitivity analysis still demonstrated good potential in terms of identifying new therapeutic targets for experimental research (Schoeberl et al., 2017; Zhao, Heuslein, et al., 2022; Zhao, Medeiros, et al., 2021). Efforts have also been devoted to the research of efficient computational methods for parameter estimation in large‐scale models (Penas et al., 2017; Villaverde et al., 2019). Such technical advances in computational algorithms and infrastructure are also very important for ensuring high levels of predictive power and translational value of systems biology‐type models in future angiogenesis research.

Therapeutic development of angiogenesis‐targeting drugs has been most successful in cancer and ocular diseases, with the vast majority of approved drugs focusing on the inhibition of VEGF‐mediated downstream signaling (Formica et al., 2021; Kong et al., 2017). On the other side, in disease areas where angiogenesis is lacking and needs to be promoted (e.g., limb ischemia/PAD, myocardial ischemia/CAD), direct therapeutic augmentation of pro‐angiogenic signaling using growth factors such as VEGF or pro‐angiogenic regulators such as HIF had failed to achieve clear efficacies in late‐phase clinical trials to date (Annex, 2013; Annex & Cooke, 2021; Mitsos et al., 2012). The overall response rates of approved anti‐angiogenics are moderate in cancer patients; however, renewed opportunities are in combinations of anti‐angiogenic and immune checkpoint inhibitors (Boucher et al., 2021; Datta et al., 2019). Although there exist many angiogenic factors in both human and mice, the tissue/whole‐body level models discussed above mostly focused on one angiogenic factor of interest (e.g., TSP‐1, EGF, VEGFs) or incorporated a generic model species that represents the overall angiogenic effect, likely due to a lack of data for model calibration. As the models showed the potentials of rendering predictions that are not intuitive when the elements of the model are viewed separately, we envision there to be a big opportunity for systems biology/systems pharmacology type models of angiogenesis signaling to play central roles in future drug development. Integrating newly hypothesized or details of known multiscale biological mechanisms and translational features into such models can further impactfully inform decision‐making in multiple stages of preclinical and clinical development (Nijsen et al., 2018). For example, incorporating omics‐derived dynamics and biological features into such mechanistic models can inform the construction of model‐based virtual patients and allow simulation of disease progression and treatment efficacy at the level of both patient populations and individuals (Lazarou et al., 2020; S. Zhang, Gong, et al., 2021). Model‐informed drug development (MIDD) as a field is also gaining ground (Marshall et al., 2019; Y. Wang, Zhu, et al., 2019). Advances in quantitative systems pharmacology (QSP) and virtual clinical trials applied to anti‐ and pro‐angiogenic drugs and drug candidates should facilitate these developments (Bai et al., 2019).

## CONFLICT OF INTEREST

The authors have declared no conflicts of interest for this article.

## AUTHOR CONTRIBUTIONS

**Yu Zhang:** Conceptualization (equal); investigation (equal); writing – original draft (equal); writing – review and editing (equal). **Hanwen Wang:** Investigation (equal); writing – original draft (equal). **Rebeca Hannah M Oliveira:** Investigation (equal); writing – original draft (equal). **Chen Zhao:** Conceptualization (lead); investigation (equal); supervision (lead); writing – original draft (equal); writing – review and editing (equal). **Aleksander S Popel:** Conceptualization (equal); funding acquisition (lead); supervision (lead); writing – review and editing (equal).

## RELATED WIREs ARTICLES

Systems medicine: Evolution of systems biology from bench to bedside

The macrophage and its role in inflammation and tissue repair: Mathematical and systems biology approaches

Systems biology of oxygen homeostasis

Context‐dependent regulation of receptor tyrosine kinases: Insights from systems biology approaches

Systems biology of pro‐angiogenic therapies targeting the VEGF system

## Data Availability

Data sharing is not applicable to this article as no new data were created or analyzed in this study.
